# Impact of Process Variables of Acetone Vapor Jet Drilling on Surface Roughness and Circularity of 3D-Printed ABS Parts: Fabrication and Studies on Thermal, Morphological, and Chemical Characterizations

**DOI:** 10.3390/polym14071367

**Published:** 2022-03-28

**Authors:** Shahbaz Juneja, Jasgurpreet Singh Chohan, Raman Kumar, Shubham Sharma, R. A. Ilyas, M. R. M. Asyraf, M. R. Razman

**Affiliations:** 1 Department of Mechanical Engineering, Chandigarh University, Gharuan, Mohali 140301, India; juneja_shehbaz@yahoo.com; 2 Mechanical Engineering Department, University Center for Research & Development, Chandigarh University, Mohali 140301, India; jaskhera@gmail.com (J.S.C.); ramankakkar@gmail.com (R.K.); 3 Department of Mechanical Engineering, IK Gujral Punjab Technical University, Main Campus-Kapurthala, Kapurthala 144603, India; 4 School of Chemical and Energy Engineering, Faculty of Engineering, Universiti Teknologi Malaysia, Johor Bahru 81310, Malaysia; ahmadilyas@utm.my; 5 Centre for Advanced Composite Materials, Universiti Teknologi Malaysia, Johor Bahru 81310, Malaysia; 6 Institute of Energy Infrastructure, Universiti Tenaga Nasional, Jalan IKRAM-UNITEN, Kajang 43000, Malaysia; asyrafriz96@gmail.com; 7 Research Centre for Sustainability Science and Governance (SGK), Institute for Environment and Development (LESTARI), Universiti Kebangsaan Malaysia (UKM), Bangi 43600, Malaysia

**Keywords:** additive manufacturing, acrylonitrile–butadiene–styrene, chemical vapor jet drilling, surface roughness and circularity, SEM analysis, DSC analysis, FTIR analysis, L_9_ Taguchi orthogonal array

## Abstract

Ever since the introduction of 3D printing, industries have seen an exponential growth in production and efficiency. Three-dimensional printing is the process of additive manufacturing (AM) in which the conventional method of material removal is challenged. Layer-on-layer deposition is the basic principle of the AM. Additive manufacturing technologies are used to create 3D-printed objects. An object is built in an additive technique by laying down successive layers of material until the object is complete. Each of these layers can be viewed as a cross-section of the item that has been lightly cut. When compared to traditional production methods, 3D printing allows the creation of complicated shapes with less material. In conventional methods, the materials go through several damages due to the tool–workpiece contact creating friction between them and the dissipated heat that damages the material. Overcoming the conventional method of machining with the help of 3D printing is a new advancement in the industries. The process involves using non-conventional methods for the machining of the parts. This research was oriented towards the chemical vapor jet drilling of the acrylonitrile–butadiene–styrene (ABS) materials. ABS materials are highly machinable and can be recycled for further usage. This paper focused on the usage of acetone as the chemical for drilling. The surface roughness and circularity of the drilled hole was taken into account for this research paper. We set up a manual experiment to run tests and get results. A vapor jet machine was designed with acetone as the core for the vapor. Various analyses were also formulated and conducted during experimentations. Surface roughness analysis provided the insight of roughness after the machining with the help of acetone vapor jet spray. SEM and micro-image parameters were also considered for more clear and advanced reports. In this research paper, DSC and FTIR analysis were performed to understand changes in the internal structure and the material properties of the ABS. Moreover, the research aimed to investigate the effect of various inputs processing parameters such as pressure, flow rate, and stand-off distance on the surface roughness and circularity of ABS workpiece material. The Taguchi L_9_ orthogonal array design was utilized to conduct tests by chemical vapor jet drilling using acetone and to evaluate the performance of the set-up while reducing the influence of interfering factors in order to provide reliable surface finish and circularity results. The results and conclusion of the research paper aimed to determine the most suitable parameters for the non-conventional acetone vapor jet drilling of the ABS material. The theoretical calculations predicted 1.64432 and 0.3289080 values of surface roughness and circularity, respectively. On the other hand, the experimental values were recorded as 1.598 for surface roughness and 0.322 for circularity. Therefore, a negligible error of 0.046 for surface roughness and 0.0031 for circularity, respectively, was noted which validate the statistical equations and the consistency of the combined vapor jet drilling process.

## 1. Introduction

**Figure 1:**
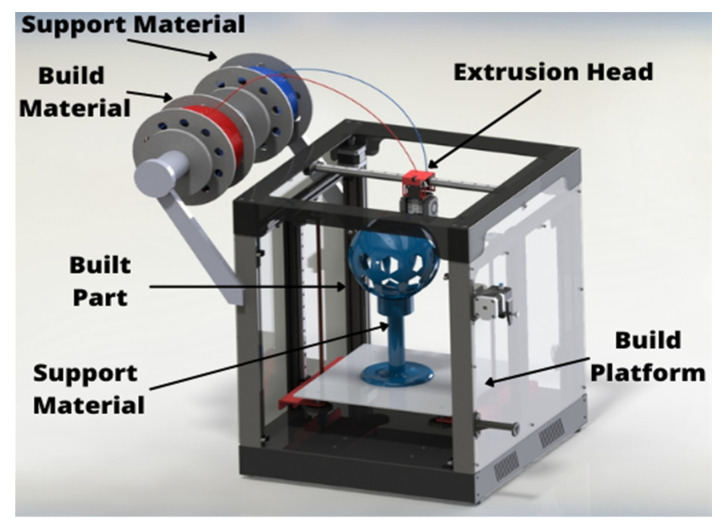
FDM apparatus set-up.

Additive manufacturing (AM) is a set that comprises a wide range of rapid tooling (RT) techniques and focuses on the production of complex geometries in an efficient way. It is a type of 3D printing which reduces time and is highly cost efficient. AM is a layered manufacturing technique which is an advancement in rapid prototyping (RP) production. It is a non-conventional method of production that eliminates the traditional manufacturing elements such as tools, machining process, jigs, and fixtures [1,2]. AM techniques such as fused deposition modeling (FDM) have their application in our day-to-day life. FDM is used in industries such as aerospace, automobile, medical, toy, construction, and marine engineering [3]. The FDM technique is a fast, inexpensive, clean, and safe method and fabricates the product in layer-by-layer deposition [4]. It is an integrated technique of RP and computer aided design (CAD) which provides flexibility to use various shapes and materials while achieving desired properties [5,6]. In FDM, the desired material is softened and put into the liquefier that melts the material and then pushed out through a nozzle to form the product as shown in Figure 1. The machine (liquefier) moves and the required shape is achieved by depositing the extruded material [7].

In recent years, the demand for mass production and reduced time and cost to manufacture high-quality products has increased, and with the demand, the need for FDM has also increased [8]. For post-manufacturing operations, certain non-conventional machining processes such as electro discharge machining (EDM), laser cutting, abrasive water jet (AWJ), and vapor finishing are used [9], as non-traditional processing does not involve point-to-point contact between tools and parts which leads to lesser wastage and wear and tear of material. These methods are an initiation of machining complex shapes with surface finishing and low cost which meets the demands of efficient and improved manufacturing requirements [10]. In FDM, materials of various grades and structures are used. Acrylonitrile–butadiene–styrene, commonly known as ABS, is used because of properties such as corrosion resistance, high grade strength, resistance to heat, and chemical moisture, and it is highly recommended for machining processes [11].

To machine or finish ABS substrates, post-processing methods include both traditional and non-conventional machining processes. Residual stresses in quenched ABS sheets were compared to equi-biaxial residual stresses by A. S. Maxwell and A. Turnbull. The layer-removal process revealed equi-biaxial and repeatable residual stresses in the polymer sheet. On the other hand, residual stresses measured via hole drilling were not equi-biaxial and did not balance across the thickness of the specimen. Finally, the layer-removal technique produced the most consistent findings for thickness residual stress measurements. The hole-drilling technique may be more versatile for assessing residual stress in complicated geometries. Moreover, Meinhard et al. also investigated the quality of a drilled hole in a carbon fiber reinforced thermoplastic laminate using a traditional drilling technique. Various imaging approaches were used to differentiate and group defects from the laminate production process as well as from the machining system, and it was discovered that burrs, rather than delamination, were the most common yet non-selective type of damage in drilled CFRTP. The most common damage pattern in thermoplastics was burr formation and fiber deflection in a plasticized matrix zone, accompanied with fiber cracks. In traditional methods, the ABS was treated with point-to-point contact due to which material deformation occurs. Traditional tools damage the surface of the ABS parts and deteriorate its finishing as heat is generated due to friction between the tool and workpiece, which further melts down the material [12]. Due to conventional processes, the cost of tools has changed and production has increased manyfold, increasing the production cost. The observation of chips revealed that material is deformed plastically [13]. Further, owing to the wastage and lack of manageability of plastic materials, 3D-printed parts are often wasted during conventional machining; hence, we need the non-traditional approach to recycle the parts. In case of such defects, recent research has been conducted to evolve a better machining process. One of the most efficient methods of machining available is the non-conventional process [14].

For post-processing non-traditional machining procedures such as electro discharge machining (EDM), laser cutting, abrasive water jet (AWJ), and vapor finishing have been utilized. Choudhury et al., for example, used a laser cutting procedure to drill holes in ABS and PMMA polymer sheets of various diameters. Keeping as independent process variables, laser power, assist gas pressure, cutting speed, and stand-off distance were considered, and the ideal values of optimal parameters were obtained at a compressed air pressure of 2.0 bar, laser force of 500 W, cutting pace of 0.6 m/min, workpiece laser distance of 5.0 mm, hole diameter of 2.0 mm, and material of PMMA. These mixes had the least amount of taper in the beginning. When ABS polymer was laser pierced, the hole was more circular at the entry than at the exit whereas in PMMA, the aperture was more round at the exit than the entry [15].

Additionally, chemical vapor processes are also used in machining. Such processes improve the surface finishing, and these techniques use methods such as chemical treatment and coating for surface improvement [16]. Machining processes such as drilling operations are functioned on 3D-printed materials. These drilling operations are carried out with non-traditional methods such as abrasive jet machining. Acetone vapor jet drilling is performed on parts to further use them as jigs, fixtures, and tools, etc. Acetone helps in the surface finishing while drilling and provides better results in terms of circularity of the drill holes [17].

In comparison to tetrahydrofuran and methyl ethyl ketone (MEK), acetone is the best liquid for treating ABS parts, whether by polishing or vapor smoothing or using it to stick parts together. Even if the solvent properties of tetrahydrofuran are the same as those of acetone, it melts at a higher temperature and evaporates at a slower rate [18,19].

As ABS material is non-biodegradable which makes the plastic wastage more prominent, it has further led to necessitated advancements in recycling of ABS material; therefore, with reference to its effect on the environment, recycling of this material is a better approach [20,21,22].

Most of the previous work revolved around drilling with lasers, abrasives, and water jets. These methods had replaced the traditional method of machining. The present study focused on alternate ways of non-conventional machining and drilling, keeping in view the recycling of ABS material. This research paper studied the effects of drilling 3D-printed parts with the help of acetone vapor. Acetone vapor jet drilling was used to perform the operation on ABS. Investigation of parameters such as surface roughness and circularity were performed. No research study has previously examined acetone drilling on 3D-printed parts; hence, this research paper may be a breakthrough for new methods of non-conventional drilling. The present study was carried out to optimize the surface roughness and circularity of the drilled holes on ABS material.

## 2. Methodology

**Figure 2:**
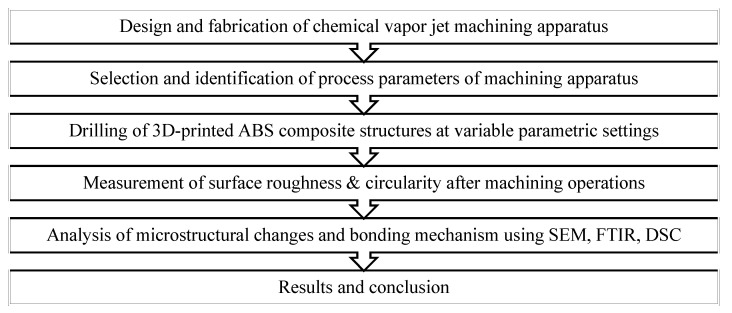
Flow chart representing methodology of this research study.

An optimization study was performed for this research to rule in the most suitable parameters for the results. This paper complied with the Taguchi technique. Acetone was applied as a surface enhancer to increase efficiency and smoothening of the treated surface. The drilling operations with acetone required a parametric evaluation of the final result. The result of this experiment allowed us to draw a conclusion on the effects of acetone vapor jet drilling on 3D-printed materials for circularity and surface finish. Methodology of the complete process is as shown below in Figure 2.

### 2.1. Experimental Set-Up

**Figure 3:**
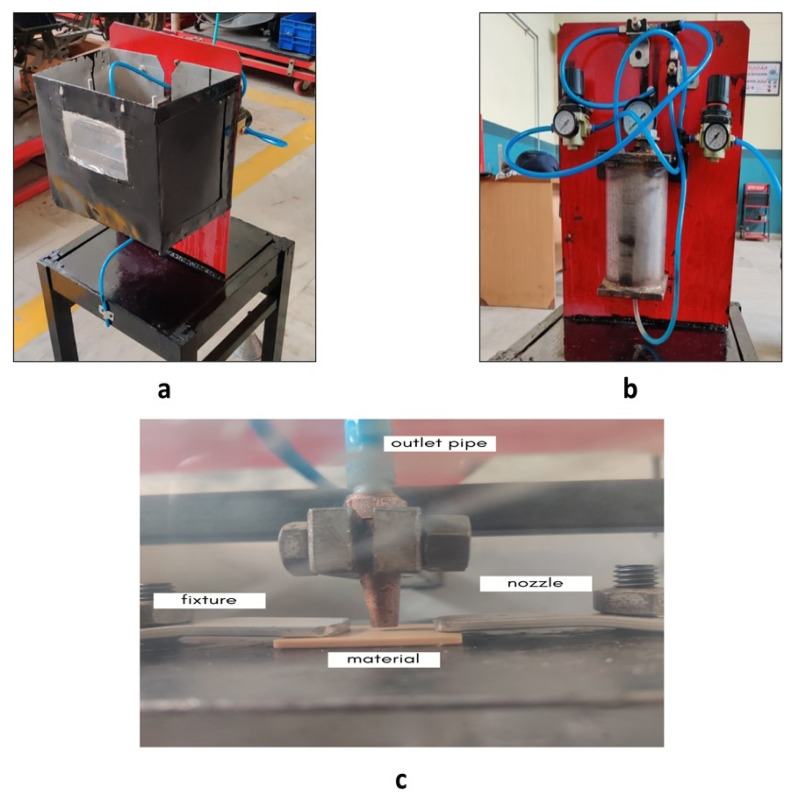
(**a**) Experimental set-up of acetone vapor jet drilling (front view). (**b**) Experimental set-up (rear view). (**c**) Nozzle of the experimental set-up to eject vapors on the workpiece.

For the present study, experiments were performed on a set-up of chemical vapor jet machine designed in-house. The machine was sectioned in two majors as shown in Figure 3a–c, of which the control flow part was the first section that comprised the compressor, mixing chamber, and gauges. The second part was called the work station section and was a buildup of control valves, nozzle, and reservoir. A high-pressure vapor of air and acetone mixture was ejected which varied as per the operating conditions of the particular experiment with the help of the flow control valve. Stand-off distance could be varied with the adjustable worktable which could be measured with the help of the filler gauge, whereas the pressure of compressed air could be adjusted with the pressure valve and measured with the help of the pressure gauge mounted on the set-up.

**Figure 4:**
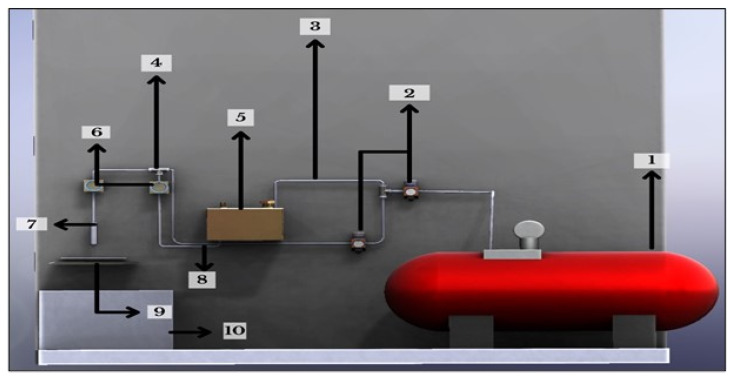
Systematic view of experimental set-up.

The parts used in the experimental set-up were based on the parameters and could easily run the set-up for various grades of ABS. The parts mentioned in Table 1 were as per the description, and the parts were shown and labeled according to the serial number of Figure 4.

### 2.2. Work Material

Acrylonitrile–butadiene–styrene (ABS) is a thermoplastic material which is used to make light and rigid parts. The quality of the ABS material makes it a more usable and optimal material. Properties such as corrosion resistance, high grade strength, high machinability, resistance to heat, chemical moisture, and various options of colors make it more viable. Impact resistance and toughness is generally considered as the choosing factor for the ABS material. The temperature range of ABS materials is between –40 °C to 100 °C, which provides a useful range for various processes and uses. ABS materials practically find their applications in most of the sectors in industries including automotive, aerospace, toy manufacturing, electrical housing, home appliances, and many others. Different properties of the ABS material are presented in Table 2.

### 2.3. Experimentation

In order to investigate the effect of process variables on the surface roughness and circularity of the ABS workpiece material, chemical vapor jet drilling using acetone under various processing variables such as pressure, flow rate, and stand-off distance was selected, considering literature review conclusions and process limitations. Selected process variables are shown in the table below.

In the final experimentation, the first phase included the preparation of the workpiece as per a required dimension (25 × 25 × 2.5 mm), which was selected as per set-up limitations. Further, workpiece was placed on the workpiece holding fixture as in setup.

Three parametric sets that included pressure of the compressed air, flow rate of acetone vapors, the distance between the workpiece and nozzle, and the machining with three different levels were used as input variables for chemical vapor jet drilling. Nozzle holding arrangement was provided just above the workpiece holding fixture and screws were provided on it to adjust the stand-off distance, i.e., distance between nozzle and workpiece. Compressed air pressure and flow rate of acetone vapors were maintained using flow-regulating valves.

Finally, when the acetone vapor jet struck on the ABS workpiece surface at a defined point, material was eroded and created a circular hole with significant surface finish around the circumference with substantial circularity. Surface roughness of the substrates was measured with the help of Mitutoyo Surftest SJ-410 (Manufactured by MITUTOYO, Aurora, IL, USA).

The drilled hole circularity was also measured to analyze drilling quality with the help of Vertex-311 (Micro-Vu’s InSpec Metrology Software, Windsor, CA, USA) which recognizes and identifies a circular shape and measures the circularity of the drilled hole. It was observed that the diameter and circularity of the hole was 2.0274 mm and 0.3855 mm, respectively.

All the experiments have been performed according to the set processing parameters as shown in Table 3 and the input variables as per Table 4.

**Figure 5:**
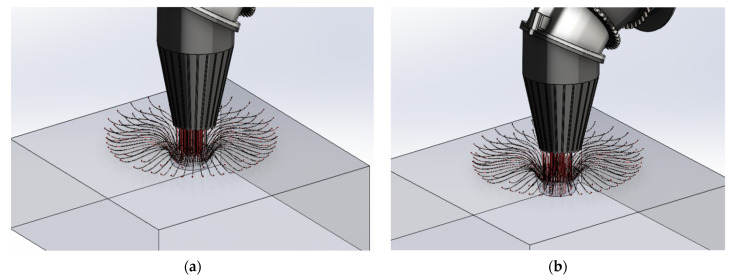

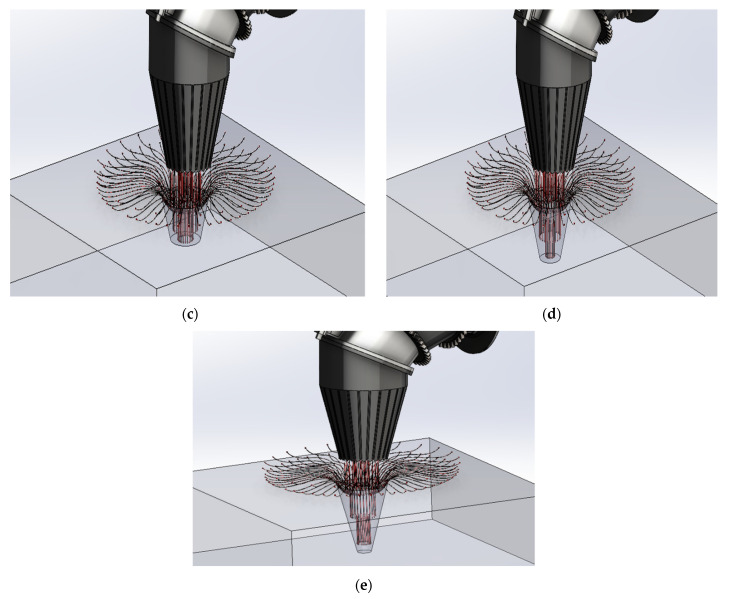
(**a**–**e**) Schematic of drilling process in progress.

Figure 5 represents the acetone vapor jet drilling process in progress with respect to different processing duration instants.

Investigation, implementation, and scheduling are required to derive meaningful and unambiguous results from measurements. The study focused on the recycling of the ABS material since 3D-printed ABS materials and related parts are non-biodegradable, so there is a significant demand to recycle ABS waste materials and its parts. To accomplish the same goal, researchers used conventional procedures, but heat and stress creation such as damage to the internal raster and rough internal surface after cooling of molten polymer layers were two main difficulties that prompted them to shift to non-traditional ways. Taking this into account, chemical vapor jet drilling was implemented. Furthermore, the research aimed to investigate the effect of various input processing parameters on surface roughness and circularity. The experimental technique of Taguchi L_9_ orthogonal array design was utilized to conduct tests using chemical vapor jet drilling and evaluate the performance of the set-up while reducing the influence of interfering factors in order to provide reliable surface finish and circularity results.

The major influence plot of SN ratios was employed to choose the best combinations of input process variables for optimal output values. Signal-to-noise ratios was utilized to assess the rank of impactful variables on output parameters. It was proposed as a technique for sorting the most and least affecting parameters between those studied. It was essential in the current study.

Increasing the responsiveness (change in surface roughness and circularity) necessitates the “smaller is better” attribute. Equation (1) as shown below was utilized for finding the SN ratio when the highest target value of a quality feature (response) was required:

S/N = −10 × log (Σ(Y^2^)/n)) (1)

where n represents the number of observations in the current investigation with n = 3.

## 3. Findings and Discussions

A total of nine trials were carried out, and a considerable improvement in surface roughness and circularity using vapor jet drilling of ABS workpieces have been investigated

### 3.1. Optimization of Acetone Vapor Jet Drilling Process Parameters for Surface Roughness and Circularity of ABS Polymer Using L_9_ Taguchi Orthogonal Array

#### 3.1.1. Influence of Variables on Surface Finish

**Figure 6:**
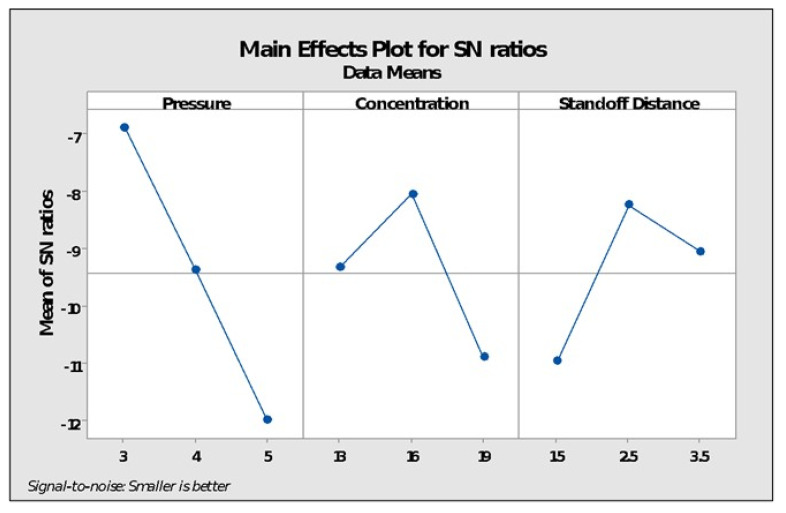
Main effect plots for SN ratios.

Figure 4 plots the factor effects on the SN ratio against various levels of each input parameter for change in surface roughness as shown in Table 5. It is evident from Figure 6 that as the pressure rose from three bars to five bars, there was a noticeable change in the SN ratio throughout. Further, with the rise in the flow rate of acetone vapors, the SN ratio rose from 13 to 16 but it began declining with further improvement in flow rate i.e., above 16. Similarly, a considerable change in the SN ratio was observed with the improvement in stand-off distance from 1.5 mm to 2.5 mm, although it declined again with further improvement.

Moreover, ANOVA was adopted for the analysis of the experimental data. The ANOVA result for surface roughness is presented in Table 6. From the table, it can be seen that input variables, namely pressure, contributed maximum influence towards surface roughness, and the flow rate and stand-off distance also had a considerable influence over the surface roughness. It was found that all the three parameters were significant as their *p* values were below 0.05 at a 95% confidence level as shown in Table 7.

**Figure 7:**
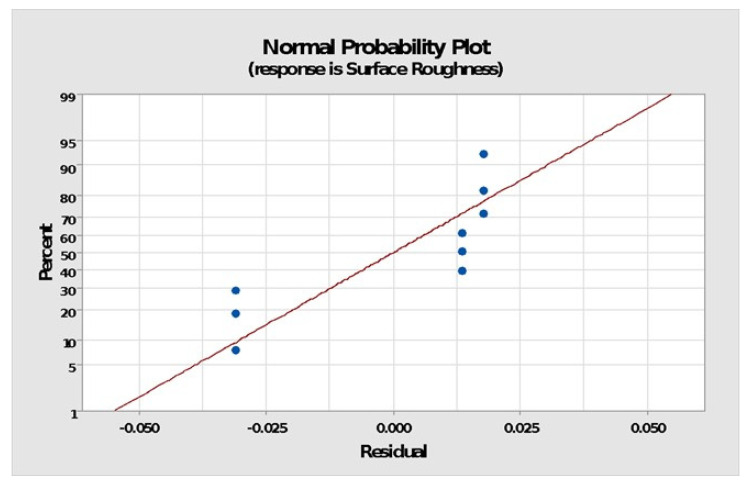
Normal probability plots of residuals.

The normal probability plots of residuals in Figure 7 show that the points for output parameters, i.e., surface roughness, often form straight lines, indicating that residuals are normally distributed. If the points deviated from a straight line, the normalcy assumption might have been considered invalid. Thus, normally distributed data demonstrate the usefulness of the Taguchi technique in creating strong designs with few errors while running fewer experiments, as discovered by earlier researchers [21,22,23,24,25,26,27,28,29,30,31,32,33,34,35].

#### 3.1.2. Influence of Variables on Circularity

**Figure 8:**
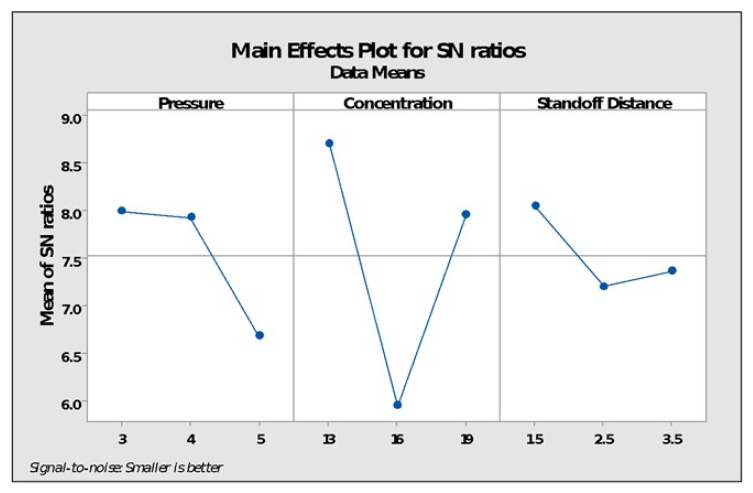
Main effect plots for SN ratios.

Figure 8 displays the factor effects on the SN ratio vs. different values of each input parameter for the change in circularity. It is clear that when the pressure rose from three to four bars, the SN ratio declined noticeably; further, pressure increase resulted in a significant decrease in the SN ratio. Furthermore, as the flow rate of acetone vapors increased from 13 to 16, the SN ratio degraded dramatically before rebounding with an additional flow rate increase. Correspondingly, a significant change in the SN ratio was detected with an increase in stand-off distance from 1.5 mm to 2.5 mm, although modest changes were observed again with further improvement. Response of circularity and SN ratio with respect to input variables is shown in Table 8.

Table 9 specified the rank of input variables with respect to their effect on circularity.

The ANOVA findings for the analysis of circularity on experimental data are shown in Table 10 below. From the table, it can be seen that the input variable of flow rate of acetone vapors contributed maximum influence (75.80%) toward circularity, with pressure also having a considerable influence (18.50%) over the circularity. Among the three criteria, stand-off distance was shown to be the least significant in terms of circularity. Pressure and flow rate were also judged to be significant because their *p* values were less than 0.05 at the 95 percent confidence level.

**Figure 9:**
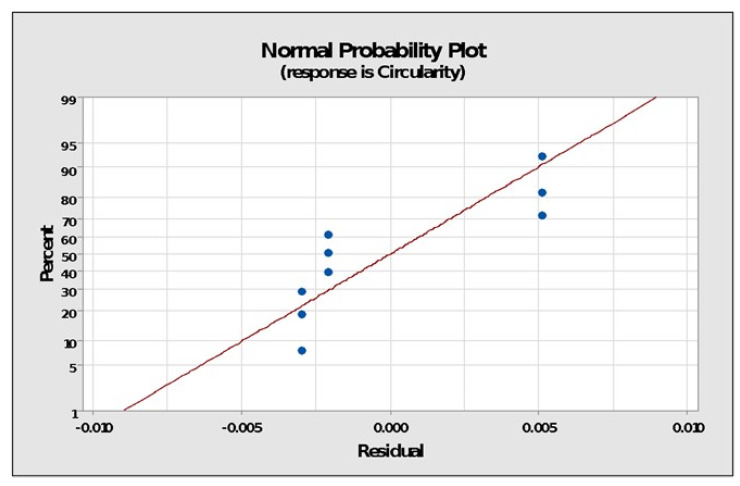
Normal probability plot.

The residual normal probability plots in Figure 9 show that the points for output parameters of circularity frequently form sharp lines, indicating that the variables were normally distributed. The normality assumption may have been judged faulty if the points diverged from a straight line.

### 3.2. SEM Microstructural Analysis

**Figure 10:**
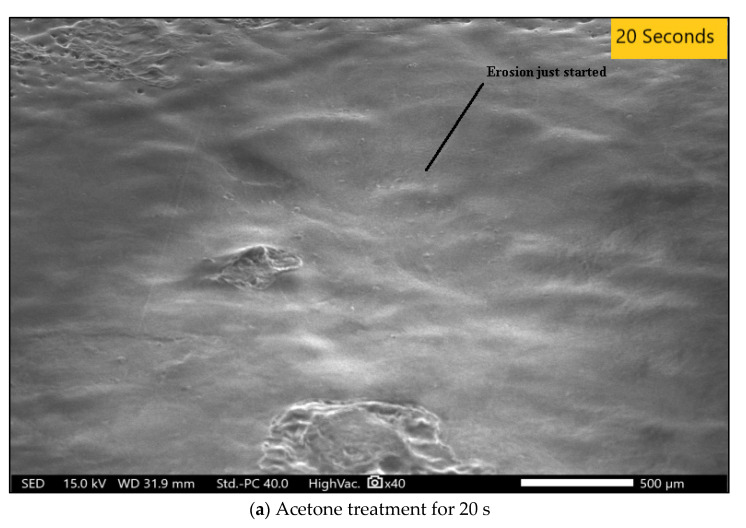

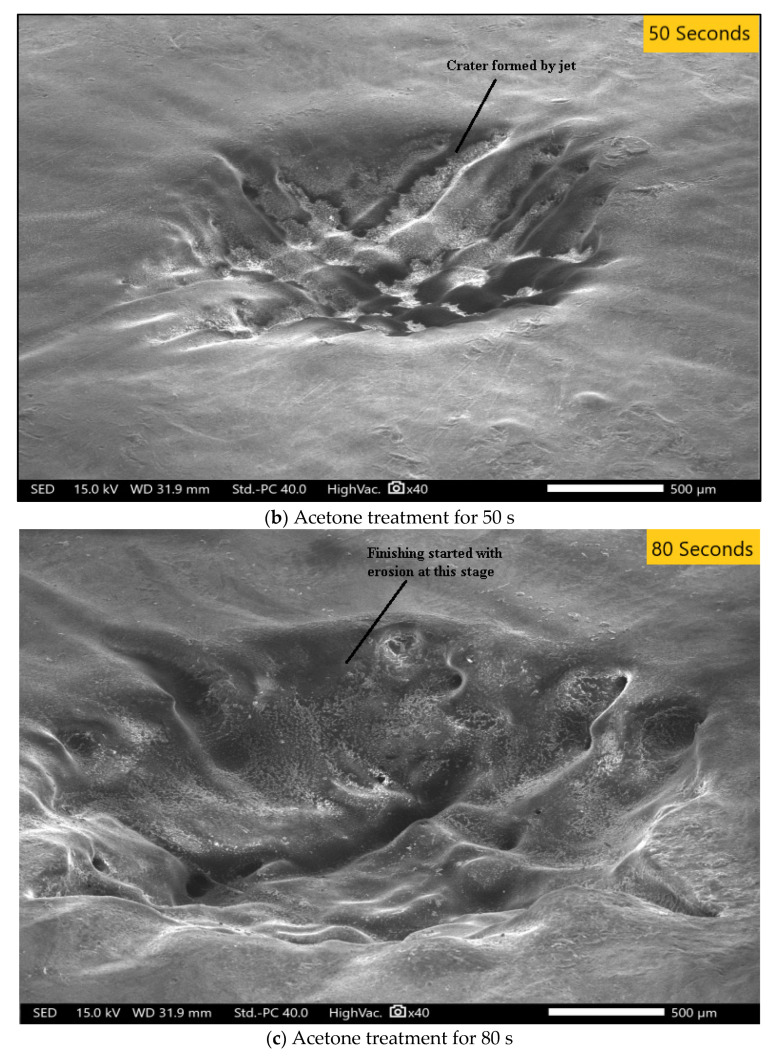
(**a**) 20 s, (**b**) 50 s, and (**c**) 80 s. Material removal SEM images after treatment with acetone.

SEM analysis, also known as scanning electron microscopy, is a method of image magnification to carry out the analysis of any material. This analysis provides data on the failures, deformations, and damages that crept in the material during various machining processes [33,34,35,36,37,38,39,40,41,42,43,44,45,46,47,48,49,50,51,52,53,54,55,56,57]. Operations such as turning, milling, and drilling carried out on a material damages the surface and internal structure, which is analyzed with SEM analysis. In our study, SEM analysis was performed on a workpiece which was processed for durations of 20 s, 50 s, or 80 s of chemical vapor jet drilling while keeping pressure at 4 bar, stand-off distance at 2 mm, and flow rate at 16 mL. SEM analysis shows the highly magnified pictorial representation of the material which makes it easier to figure out the changes, surface roughness, and the circularity of the experimental data. In acetone vapor jet drilling used in our research, the images obtained from the SEM data as shown in Figure 4 allowed us to conclude that the rate of material removal was directly proportional to the time of drilling operation. If the operation was performed for longer durations, the removed material was higher in quantity. The depth as obtained from the data was 0.27 mm of material removed for every 20 s of operation. In the experimentation, the ABS material used as workpiece was of 2.5 mm of thickness. Wave-like patterns were introduced on the surface after the initial 20 s of operation as shown in Figure 10a. The depth was 0.27 mm for the first operation performed for 20 s, 1.38 mm after the 50 s of drilling operation, and 2.20 mm after the final operation of 80 s. In the second operation, the depth of the cut was clearly visible and after the final operation, the surface of the material could be seen more smoothly and clearly with the circularity of the cut being rounder and finer. SEM analysis provided data on circularity of the hole. The width and depth of the cut after the 20 s operation was 1.945 mm and 2.127 mm, respectively. Similarly, for 50 s of operation, the circularity of the hole was much more evenly distributed with a width of 2.177 mm and a cut depth of 2.087 mm. In the final stage of 80 s of operation, a width of 3.035 mm was observed along with a depth of 3.016 mm. The circularity for the 80 s operation was better than the other optimization.

**Figure 11:**
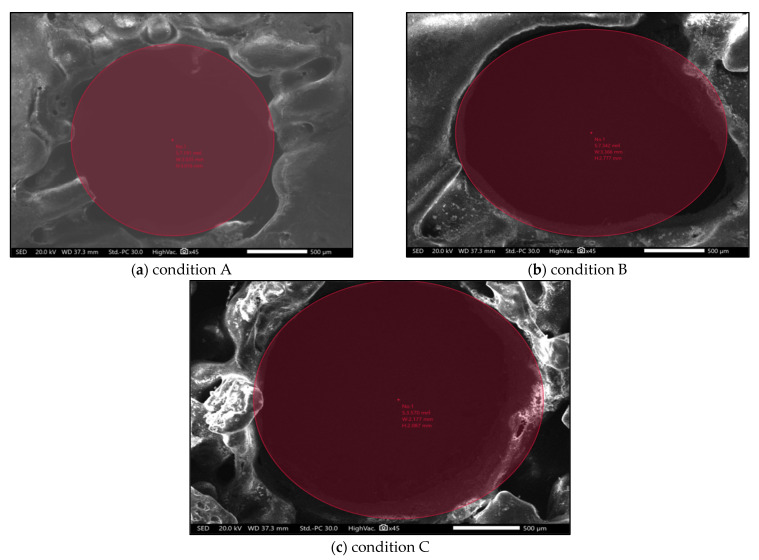
(**a**) SEM image with condition A of acetone vapor jet treatment. (**b**) SEM image with condition B of acetone vapor jet treatment. (**c**) SEM image with condition F of acetone vapor jet treatment.

Figure 10a reveals that after 20 s of acetone treatment, the material removal was significantly reduced. There was no significant change and the surface was quite rough. Figure 10b shows that the effects were enlarged and could be seen because the duration of the acetone treatment was increased to 50 s. The material removal and the depth of the cut was greater as shown in Table 11. For the final analysis, SEM images were taken for experimental condition A, B, and C shown in Figure 11. After comparison of Figure 10a–c, it can be clearly seen that condition A, the least level of all the input variables, provided the best circularity result which was also verified from circularity response values. However, condition B provided the poorest circularity result.

### 3.3. Differential Scanning Calorimetry

**Figure 12:**
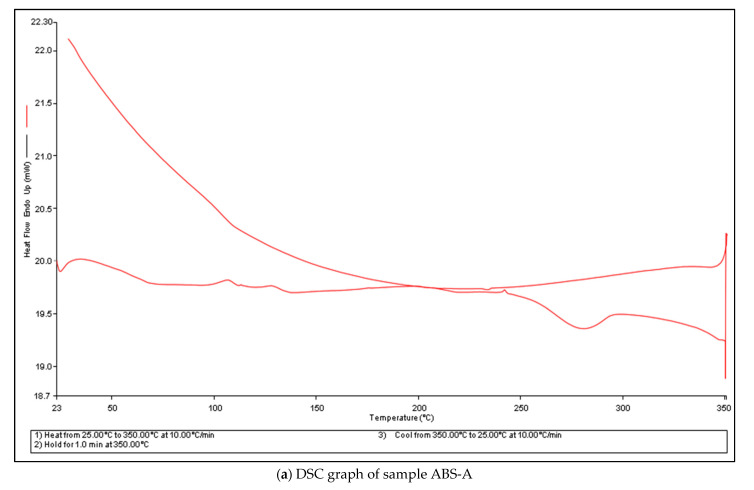

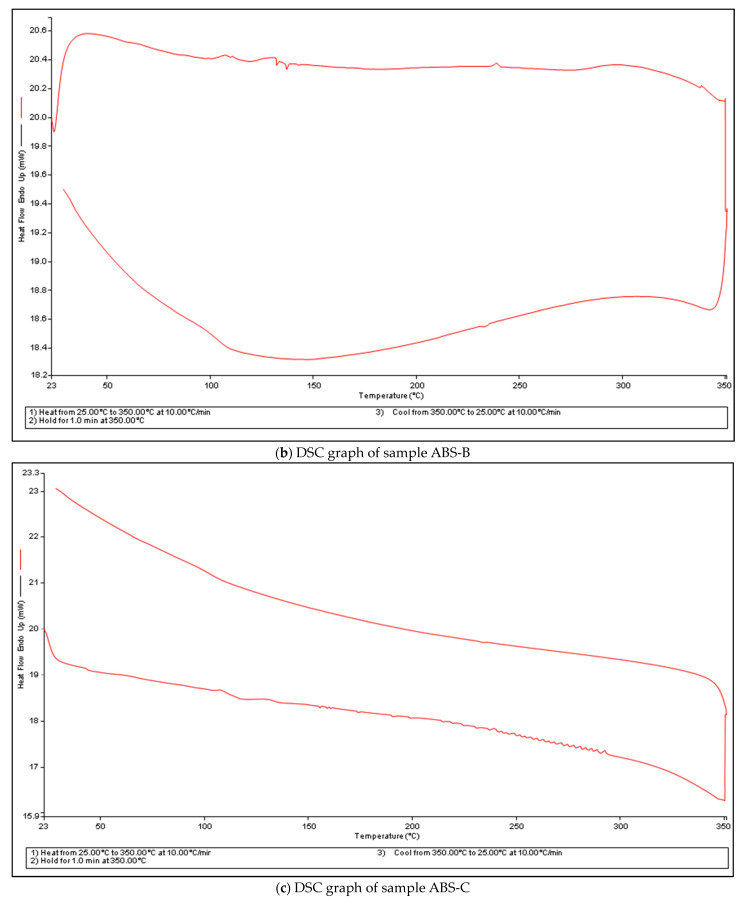

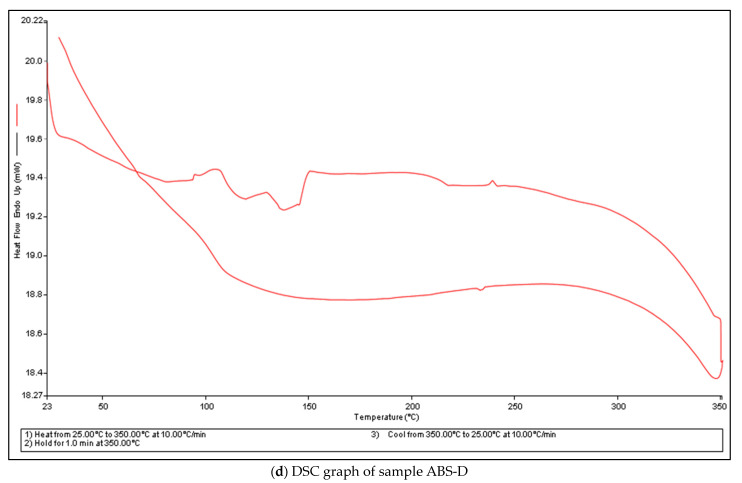
DSC graphs for different samples (**a–d**).

DSC, differential scanning calorimetry, is a thermal analysis method of changes in the material due to changes in the heat and temperature. This method works on the principle of effects due to heat in and out, that is, the heat absorbed and emitted from the material, and the changes are then observed under the controlled temperature program. The observations are made on the pattern of changes in the physical properties with respect to time and temperature. The changes in the melting point and crystallization point are measured along with similar ranges. DSC measures the heat effect and changes with reference to a standard material. In our experimentation, the material underwent a three-level heating and cooling process as standardized for the DSC method [55,56,57,58,59,60,61,62,63]. The three-level process involved heating at room temperature from 25 °C to 350 °C at a rate of 10 °C per minute as shown in the Figure 12a–d. In the second level of the process, the material was maintained at the temperature of 350 °C for 1 min, and in the final level of DSC, the material was cooled at a rate of 10 °C from the temperature of 350 °C to 25 °C. The materials that we used were samples of ABS, namely ABS-A, ABS-B, ABS-C, and ABS-D as descripted in Table 12.

Heat is absorbed or emitted from the material and hence changes the temperature and physical properties while going through a phase transition. The temperature of the phase transition is called glass transition temperature (Tg), at which the material shows macromolecular properties. The DSC graph reveals that the glass transition temperature for the materials was 104 °C. Specific heat capacity (Cp) is the amount of heat required for raising the temperature of unit mass by 1 °C. The specific heat capacity is a dependent quantity on temperature which rises in proportion [58,59,60,61,62,63,64,65,66,67]. While raising the temperature to the ABS material by imparting heat to it, the material starts to melt, also known as melting temperature (T_m_). The DSC analysis graph reveals that the materials after the treatment of acetone vapor jet drilling concluded to various melting temperature at 238.85 °C, 227.41 °C, and 239.03 °C as shown in Table 13. The T_m_, or the melting temperature, provide insights about how a material will function under high temperature. The T_m_ denotes the melting of the material, which means that the physical and chemical properties of the materials are changed. Hence, with the help of DSC analysis, we can also obtain safe working temperatures and parameters for any material. In our analysis, the temperature raised in ABS materials was due to acetone vapors that did not match the T_m_. This analysis is consistent with the reason for opting out of the acetone vapor jet and acetone chemical which is that it does not regulate or change the physical and chemical properties of acetone; hence, these materials can function at its fullest.

### 3.4. Fourier Transform Infrared Spectroscopy

**Figure 13:**
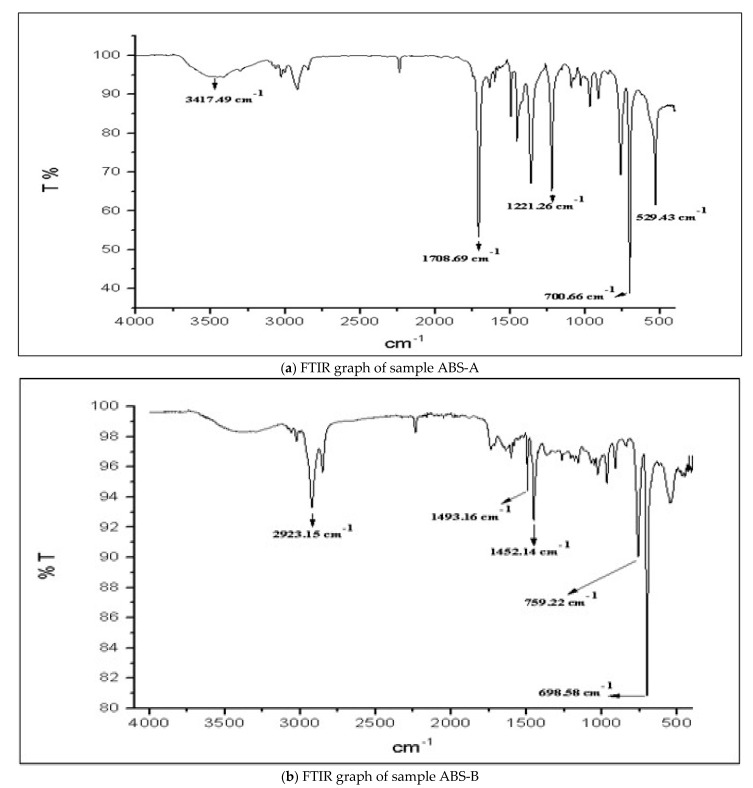

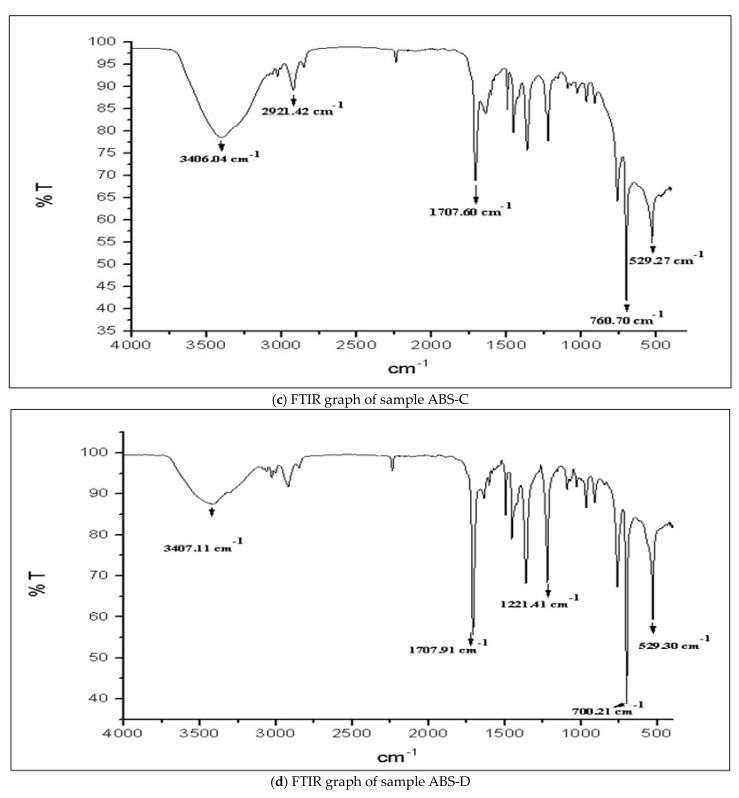
FTIR graphs 9 (**a**–**d**).

A material if passed through the electromagnetic spectrum either absorbs the spectrum or lets the spectrum pass through it. In FTIR, the infrared spectrum absorption or emittance decides the presence of groups in various materials. The spectroscopy concludes the amount of transmittance which further evaluates the amount of different functional substances and groups present. This experimentation was set up on Perkin-Nicolet Nexus 470. The basic principle behind FTIR is the infrared transmittance and emittance through the materials. Lights of various wavelengths and spectra are passed through the material. The materials then, as per their structure and properties, absorb or emit some or all of the light. These data were recorded and analyzed using the information presented in Table 14 below. The range of the FTIR spectrum was about 400 cm^−1^ to 4000 cm^−1^. The data revealed that high absorption of the light led to the presence of a high number of bonds in the material. FTIR analysis was depicted graphically and the curves of the graphs were used to conclude the data (Figure 13). The curve represents various wave numbers and the transmittance of the light through the material at specific wave numbers.

In our experimental set-up, the ABS materials showed vivid curves at different wavelengths after treatment with acetone. The data were used to draw resemblance of bond numbers and material property changes in the ABS material. The percentage of transmittance was directly proportional to the presence of several elements and bonds. In the graphs given below, we can observe a broad spectrum curve between 3600 cm^−1^ and 3200 cm^−1^. These curves represent the presence of hydroxyl groups (–OH). There are several minified curves at around 3000 cm^−1^ to 2800 cm^−1^. This minified broad spectrum is also a part of the –OH groups bond in the material [68,69,70,71,72,73,74,75]. The other functional group present in the ABS material after the treatment with acetone was the aromatic compounds bond, as we can see peaks of the curves at around 1600 cm^−1^ to 1400 cm^−1^. There are several peaks with a high level of transmittance at the end of the wave. The wave numbers between 1000 cm^−1^ to 600 cm^−1^ show peaks and needles of transmittance of about 92% as shown in Table 15. Hence, the FTIR analysis concluded the presence of majorly –OH, aromatic compounds, and CO bonds in the ABS material after the treatment with acetone.

### 3.5. Validation of Optimized Factors

The influence of various factors on the response, as stated earlier, is based on the results of quantitative descriptive statistical analysis. It is necessary to compare statistical results. Calculations based on experimental values obtained at optimal conditions accepted the vapor jet drilling for recycling of manufactured parts. Confirmatory investigations were carried out in order to substantiate the findings.

The optimal settings are as follows: A1B2C2 for the greatest change in surface roughness and A1B1C1 for the circularity as obtained from the response table for signal-to-noise ratios i.e., Table 6 and Table 8. A measurement recurrence technique was implemented, and the investigational results were compared to the theoretically predicted values [67,68,69,70,71,72,73,74,75,76,77]. The values of the SN ratios for surface roughness and circularity could be computed under ideal conditions using Equations:

Ƞ_opt =_ M_ΔRa +_ (M_A1_ − M_ΔRa_) + (M_B2_ − M_ΔRa_) + (M_C2_ − M_ΔRa_) (2)

here, M_ΔRa_ = mean of SN ratios of _ΔRa_= −84.7839 (mean of column No. 6 in Table 5).

Substitute optimal values (M_A1_, M_B2_, M_C2_) from Table 6 for surface roughness in Equation (2). This gives:

Ƞ_opt =_ −9.42043 + (−6.879 − (−9.42043)) + (−8.0446 − 8(−9.42043)) + (−8.237 − (−9.42043)) (3)

Ƞ_opt_ = −4.31974

The corresponding values of surface roughness can be calculated using formula:

Y^2^ _opt_ = 10 ^−^^ƞopt/10^ (4)

Equation (4) was derived from Equation (1) where Y_opt_ = 1.64432 is optimal response surface roughness achieved at optimized parameter settings A_1_B_2_C_2_.

Similarly, the optimal value of SN ratios (Ƞ_opt_) for circularity is given as:

Ƞ_opt =_ M_ΔC_ + (M_A1_ − M_ΔC_) + (M_B1_ − M_ΔC_) + (M_C1_ − M_ΔC_) (5)

here, M_ΔC_ = mean of SN ratios of _ΔC_ = 67.79021 (mean of column No. 6 in Table 8).

Substitute optimal values (M_A1_, M_B1_, M_C1_) from Table 9 for circularity in Equation (4). This gives:

Ƞ_opt =_ 7.532245 + (7.988 − 7.532245) + (8.695 − 7.532245) + (8.040 − 7.532245) (6)

Ƞ_opt_ = 9.65851

Using Equation (2), Y_opt_ = 0.3289080 which is an optimal value of circularity achieved at optimized parameter settings A_1_B_1_C_1_.

The theoretical calculations predicted values of 1.64432 and 0.3289080 for surface roughness and circularity, respectively. On the other hand, the experimental values were recorded as 1.598 for surface roughness and 0.322 for circularity. Therefore, a negligible error of 0.046 for surface roughness and 0.0031 for circularity, respectively, was noted which validate the statistical equations and the consistency of the combined vapor jet drilling process.

## 4. Conclusions

A comprehensive examination of the machining impacts of acetone vapor jet on the surface finishing and circularity of 3D-printed ABS material was carried out. The proposed procedure resulted in improved material surface finish and circularity. Major inferences are given below:

- Taguchi analysis revealed that pressure has maximum impact on surface roughness. The lower pressure of the vapor jet (3 bar) and a medium setting of flow rate (16 mL/min.) and 2.5 mm results in a better finish.
- Circularity was significantly influenced by vapor concentration and as per SN ratio graphs, all the parameters must be maintained at lower levels for yielding better results.
- The SEM and micro-image analyses showed that the time of acetone treatment enhances drilling quality and efficiency. The longer the surface was exposed to acetone, the more material it eliminated and the smoother it became.
- Because the rate of material removal rose with time, the time duration of vapor machining was directly related to the overall improvement in machining effects and drilling of the parts.
- The surface roughness improved over time, and the acetone vapor treatment could also be employed in the treatment of recyclable materials.
- Waste industrial equipment such as jigs and fixtures could be recycled and reused; other electronics products could also be recycled for further use.
- According to DSC, the typical glass transition temperature (Tg) is 105 °C. For vapor treated samples, a high melting temperature and melting enthalpy were observed, resulting in higher thermal resistance. The presence of hydroxyl (OH) and aromatic chemicals in the ABS material was revealed by FTIR. More research is necessary on machining and drilling as well as surface smoothening with non-traditional procedures and chemicals.
- Successfully investigated the effects of chemical treatment on dimensional stability, hole and surface quality, regularity, and smoothness after drilling of the parts.

## Figures and Tables

**Table 1:** Description of parts used in experimental set-up.

S. NO.	Material	Description
1	Compressor	Increases the working pressure to 12 bar.
2	Pressure gauge	Maintains and regulates the pressure. Maximum pressure is 10 kgF/cm^2^ or 140 psi.
3	Inlet pipe	Functions as a pathway for compressed air.
4	T-joint	For joining of pipes, inlets and outlets.
5	Mixing chamber	Mixing of vapors and acetone to create homogeneous solution. Mild steel material build and 2.5 L volume.
6	Flow control valve	Regulates the flow rate of the fluid. Maximum pressure is 10 kgF/cm^2^ or 140 psi.
7	Nozzle	It is the outlet for high-pressure spray of liquids.
8	Outlet pipe	Connects the mixing chamber to nozzle and flow control.
9	Workpiece	The workpiece stand to hold the job for the experimentation.
10	Reservoir	Storage unit to collect and recycle extra wasted vapor. About 40% of unused acetone vapor is collected here.

**Table 2:** Physical, mechanical & thermal properties of ABS.

Physical Properties		
Property	Extruded	Moulded
Density (g/cm^3^)	0.350–1.26	1.02–1.17
Moisture absorption at equilibrium (%)	0.150–0.200	0.000–0.200
Viscosity (cP) (temperature 240–260 °C)	155,000–255,000	1.16 × 10^6^–1.52 × 10^6^
Linear mould shrinkage (cm/cm)	0.00240–0.0120	0.00200–0.00900
**Mechanical properties**		
Hardness Rockwell R	90.0–121	68.0–115
Tensile strength, ultimate (MPa)	27.0–52.0	28.0–49.0
Tensile strength, yield (MPa)	20.0–62.0	13.0–65.0
Modulus of elasticity (GPa)	1.52–6.10	1.00–2.65
Elongation at yield (%)	0.620–30.0	1.70–6.00
Flexural modulus (GPa)	1.20–5.50	1.61–5.90
Flexural yield strength (MPa)	28.3–81.0	40.0–111
Charpy impact, notched (J/cm²)	0.900–5.00	0.400–14.0
Izod impact, notched (J/cm)	0.380–5.87	0.100–6.40
**Thermal properties**		
Thermal conductivity (W/m-K)	0.150–0.200	0.128–0.200
Coefficient of thermal expansion, linear (μm/m- °C)	68.0–110	0.800–155
Glass transition temperature (°C)	108–109	105–109

**Table 3:** Processing variables and their different levels.

Processing Parameters	Symbol	Level		
		1	2	3
Pressure (Bars)	A	3	4	5
Flow rate (ml/min)	B	13	16	19
Stand-off distance (mm)	C	1.5	2.5	3.5

**Table 4:** Values of input variables.

Condition	Pressure (Bars)	Flow Rate (mL/min)	Stand-off Distance (mm)
A	3	13	1.5
B	3	16	2.5
C	3	19	3.5
D	4	13	2.5
E	4	16	3.5
F	4	19	1.5
G	5	13	3.5
H	5	16	1.5
I	5	19	2.5

**Table 5:** Surface roughness responses.

S. No.	Pressure	Flow Rate	Stand-Off Distance	Surface Roughness	SNRA1
1	3	13	1.5	2.664	−8.51068
2	3	16	2.5	1.598	−4.07154
3	3	19	3.5	2.528	−8.05554
4	4	13	2.5	2.56	−8.1648
5	4	16	3.5	2.461	−7.82223
6	4	19	1.5	4.056	−12.162
7	5	13	3.5	3.663	−11.2767
8	5	16	1.5	4.095	−12.2451
9	5	19	2.5	4.205	−12.4753

**Table 6:** Response table for signal-to-noise ratios. Smaller is better.

Level	Pressure	Flow Rate	Stand-Off Distance
1	−6.879	−9.317	−10.973
2	−9.383	−8.046	−8.237
3	−11.999	−10.898	−9.052
Delta	5.120	2.851	2.735
Rank	1	2	3

**Table 7:** ANOVA results for surface roughness.

Source	DF	Seq SS	Contribution	Adj SS	Adj MS	F-Value	*p*-Value
Pressure	2	4.47992	64.79%	4.47992	2.23996	1014.73	0.001
Flow rate	2	1.23312	17.83%	1.23312	0.61656	279.31	0.004
Stand-off Distance	2	1.19715	17.31%	1.19715	0.59858	271.16	0.004
Error	2	0.00441	0.06%	0.00441	0.00221		
Total	8	6.91462	100.00%				

**Table 8:** Circularity responses.

S. No.	Pressure	Flow Rate	Stand-off Distance	Circularity	SNRA1
1	3	13	1.5	0.332	9.577238
2	3	16	2.5	0.4951	6.106141
3	3	19	3.5	0.3855	8.279512
4	4	13	2.5	0.3629	8.804261
5	4	16	3.5	0.496	6.090366
6	4	19	1.5	0.3598	8.878777
7	5	13	3.5	0.4119	7.704164
8	5	16	1.5	0.521	5.663246
9	5	19	2.5	0.4631	6.686504

**Table 9:** Response table for signal-to-noise ratios. Smaller is better.

Level	Pressure	Flow Rate	Stand-Off Distance
1	7.988	8.695	8.040
2	7.924	5.953	7.199
3	6.685	7.948	7.358
Delta	1.303	2.742	0.841
Rank	2	1	3

**Table 10:** ANOVA results for circularity.

Source	DF	Seq SS	Contribution	Adj SS	Adj MS	F-Value	*p*-Value
Pressure	2	0.007234	18.50%	0.007234	0.003617	60.95	0.016
Flow rate	2	0.029647	75.80%	0.029647	0.014824	249.80	0.004
Stand-off Distance	2	0.002110	5.40%	0.002110	0.001055	17.78	0.053
Error	2	0.000119	0.30%	0.000119	0.000059		
Total	8	0.039110	100.00%				

**Table 11:** Material removal results.

S. NO.	Duration (s)	Depth (mm)	Material Removal (mm^3^)
1	20	0.27	0.2660
2	50	1.38	2.1548
3	80	2.20	6.5702

**Table 12:** ABS sample details for DSC and FTIR.

Material	Time Duration
ABS-A	Unprocessed material for reference
ABS-B	Treated for 2 s
ABS-C	Treated for 15 s
ABS-D	Treated for 25 s

**Table 13:** Optimization of DSC graph.

Work Piece	Weight (mg)	T_g_ (°C)	Cp (J/K)	T_m_ (°C)	Rm (°C)	Rc (°C)
ABS-A	1.300	104	0.216	128.03	98–134	256–295
ABS-B	1.600	104	0.120	238.85	102–113	132–138
ABS-C	2.500	105	0.203	227.41	104–110	110–117
ABS-D	2.600	106	0.198	239.03	237–241	107–148

**Table 14:** Presence of functional group in FTIR analysis.

SL. No.	Frequency Range (cm^−1^)	Functional Group
1	3854	O–H strectching vibrations
2	3587.12	Phenols
3	3373–3422	Bonded N–H/C–H/O–H stretching of amines and amides
4	2918.2–2954	C–H
5	2500–3300	Carboxyl acid
6	2322.8–2138.1	C–N
7	2047.30	Silicon compounds
8	1733.59	Ketones
9	1405–1445	Alkanes
10	1421–1415	C–O/C–H bending
11	1382–1036	C–O
12	1215–1325	Alkyl ketones
13	1020–1220	Alkyl amines
14	1026	Vibration of C–O in alcohol hydroxyl group
15	469	Alkyl halides

**Table 15:** Bonding table of FTIR.

W. N. (Wavenumber) (cm^−1^)	Bonds Present	ABS-A	ABS-B	ABS-C	ABS-D
3600–2800	–OH	98%	77%	80%	85%
1550–1400	Aromatic compounds	92%	65%	60%	65%
1000–600	C–O	81%	42%	38%	35%

## Data Availability

No data were used to support this study.

## References

[1] Kuo C.-C., Tasi Y.-R., Chen M.-Y., Yan Z.-Y. (2021). Development of a cost-effective technique for batch production of precision wax patterns using 3D optical inspection and rapid tooling technologies. Int. J. Adv. Manuf. Technol..

[2] Majeed A., Zhang Y., Ren S., Lv J., Peng T., Waqar S., Yin E. (2020). A big data-driven framework for sustainable and smart additive manufacturing. Robot. Comput. Manuf..

[3] Boyard N., Christmann O., Rivette M., Kerbrat O., Richir S. (2018). Support optimization for additive manufacturing: Application to FDM. Rapid Prototyp. J..

[4] Yadav D., Chhabra D., Garg R.K., Ahlawat A., Phogat A. (2019). Optimization of FDM 3D printing process parameters for multi-material using artificial neural network. Mater. Today Proc..

[5] Daminabo S.C., Goel S., Grammatikos S.A., Nezhad H.Y., Thakur V.K. (2020). Fused deposition modeling-based additive manufacturing (3D printing): Techniques for polymer material systems. Mater. Today Chem..

[6] Peng A., Xiao X., Yue R. (2014). Process parameter optimization for fused deposition modeling using response surface methodology combined with fuzzy inference system. Int. J. Adv. Manuf. Technol..

[7] Peng X., Kong L., Fuh J., Wang H. (2021). A Review of Post-Processing Technologies in Additive Manufacturing. J. Manuf. Mater. Process..

[8] Sheoran A.J., Kumar H. (2019). Fused Deposition modeling process parameters optimization and effect on mechanical properties and part quality: Review and reflection on present research. Mater. Today Proc..

[9] Huang W.-B., Zhang L.-W., Li W.-L., Sun J.-S., Liang W.-J., Song X.-J., Mao X.-D., Wang Z.-J. Various Types and Applications of Additive Manufacturing. Proceedings of the 2019 International Conference on Applied Mathematics, Modeling, Simulation and Optimization (AMMSO 2019).

[10] Paul L., Babu J., Davim J.P. (2020). Non-conventional Micro-machining Processes. Materials Forming, Machining and Post Processing.

[11] Pérez M., Medina-Sánchez G., García-Collado A., Gupta M., Carou D. (2018). Surface Quality Enhancement of Fused Deposition Modeling (FDM) Printed Samples Based on the Selection of Critical Printing Parameters. Materials.

[12] Teti R. (2002). Machining of Composite Materials. CIRP Ann..

[13] Hocheng H., Puw H.Y. (1993). Machinability of Fiber-ReinforcedThermoplastics in Drilling. J. Eng. Mater. Technol..

[14] Thakur S., Verma A., Sharma B., Chaudhary J., Tamulevicius S., Thakur V.K. (2018). Recent developments in recycling of polystyrene based plastics. Curr. Opin. Green Sustain. Chem..

[15] Müller F., Monaghan J. (2000). Non-conventional machining of particle reinforced metal matrix composite. Int. J. Mach. Tools Manuf..

[16] Galantucci L., Lavecchia F., Percoco G. (2010). Quantitative analysis of a chemical treatment to reduce roughness of parts fabricated using fused deposition modeling. CIRP Ann..

[17] Colpani A., Fiorentino A., Ceretti E. (2019). Characterization of chemical surface finishing with cold acetone vapours on ABS parts fabricated by FDM. Prod. Eng..

[18] Chaudhari A.A., Godase A.M., Ravindra J., Abhijit N. (2017). Acetone Vapor Smoothing: A Postprocessing Method for 3D Printed ABS Parts. Int. J. Res. Sci. Innov..

[19] Puerta A.P.V., Fernandez-Vidal S., Batista M., Girot F. (2019). Fused deposition modelling interfacial and interlayer bonding in PLA post-processed parts. Rapid Prototyp. J..

[20] Isaev A., Grechishnikov V., Pivkin P., Mihail K., Ilyuhin Y., Vorotnikov A. (2016). Machining of Thin-walled Parts Produced by Additive Manufacturing Technologies. Procedia CIRP.

[21] Kim J.K., Kang C.K. (1995). Basic Studies on Recycling of ABS Resin. Polym. Technol. Eng..

[22] Pakkanen J.A., Manfredi D., Minetola P., Iuliano L. (2017). About the use of recycled or biodegradable filaments for sustainability of 3D printing: State of the art and research oppor-tunities. Smart Innov. Syst. Technol..

[23] Roslan Z., Ramli Z., Razman M., Asyraf M., Ishak M., Ilyas R., Nurazzi N. (2021). Reflections on Local Community Identity by Evaluating Heritage Sustainability Protection in Jugra, Selangor, Malaysia. Sustainability.

[24] Asyraf M., Ishak M., Norrrahim M., Nurazzi N., Shazleen S., Ilyas R., Rafidah M., Razman M. (2021). Recent advances of thermal properties of sugar palm lignocellulosic fibre reinforced polymer composites. Int. J. Biol. Macromol..

[25] Asyraf M.R.M., Rafidah M., Azrina A., Razman M.R. (2021). Dynamic mechanical behaviour of kenaf cellulosic fibre biocomposites: A comprehensive review on chemical treatments. Cellulose.

[26] Ali S., Razman M., Awang A., Asyraf M., Ishak M., Ilyas R., Lawrence R. (2021). Critical Determinants of Household Electricity Consumption in a Rapidly Growing City. Sustainability.

[27] Asyraf M., Ishak M., Syamsir A., Nurazzi N., Sabaruddin F., Shazleen S., Norrrahim M., Rafidah M., Ilyas R., Rashid M.Z.A. (2021). Mechanical properties of oil palm fibre-reinforced polymer composites: A review. J. Mater. Res. Technol..

[28] Alias A.H., Norizan M.N., Sabaruddin F.A., Asyraf M.R.M., Norrrahim M.N.F., Ilyas A.R., Kuzmin A.M., Rayung M., Shazleen S.S., Nazrin A. (2021). Hybridization of MMT/Lignocellulosic Fiber Reinforced Polymer Nanocomposites for Structural Applications: A Review. Coatings.

[29] Ilyas R.A., Zuhri M.Y.M., Norrrahim M.N.F., Misenan M.S.M., Jenol M.A., Samsudin S.A., Nurazzi N.M., Asyraf M.R.M., Supian A.B.M., Bangar S.P. (2022). Natural Fiber-Reinforced Polycaprolactone Green and Hybrid Biocomposites for Various Advanced Applications. Polymers.

[30] Ilyas R.A., Zuhri M.Y.M., Aisyah H.A., Asyraf M.R.M., Hassan S.A., Zainudin E.S., Sapuan S.M., Sharma S., Bangar S.P., Jumaidin R. (2022). Natural Fiber-Reinforced Polylactic Acid, Polylactic Acid Blends and Their Composites for Advanced Applications. Polymers.

[31] Nurazzi N.M., Asyraf M.R.M., Rayung M., Norrrahim M.N.F., Shazleen S.S., Rani M.S.A., Shafi A.R., Aisyah H.A., Radzi M.H.M., Sabaruddin F.A. (2021). Thermogravimetric Analysis Properties of Cellulosic Natural Fiber Polymer Composites: A Review on Influence of Chemical Treatments. Polymers.

[32] Nurazzi N.M., Asyraf M.R.M., Fatimah Athiyah S., Shazleen S.S., Rafiqah S.A., Harussani M.M., Kamarudin S.H., Razman M.R., Rahmah M., Zainudin E.S. (2021). A Review on Mechanical Performance of Hybrid Natural Fiber Polymer Composites for Structural Applications. Polymers.

[33] Asyraf M.R.M., Ishak M.R., Sapuan S.M., Yidris N., Ilyas R.A., Rafidah M., Razman M.R. (2020). Potential Application of Green Composites for Cross Arm Component in Transmission Tower: A Brief Review. Int. J. Polym. Sci..

[34] Alsubari S., Zuhri M.Y.M., Sapuan S.M., Ishak M.R., Ilyas R.A., Asyraf M.R.M. (2021). Potential of Natural Fiber Reinforced Polymer Composites in Sandwich Structures: A Review on Its Mechanical Properties. Polymers.

[35] Asyraf M.R.M., Ishak M.R., Syamsir A., Amir A.L., Nurazzi N.M., Norrrahim M.N.F., Asrofi M., Rafidah M., Ilyas R.A., Rashid M.Z.A. (2022). Filament-wound glass-fibre reinforced polymer composites: Potential applications for cross arm structure in transmission towers. Polym. Bull..

[36] Sharma S., Patyal V., Sudhakara P., Singh J., Petru M., Ilyas R.A. (2021). Mechanical, morphological, and fracture-deformation behavior of MWCNTs-reinforced (Al–Cu–Mg–T351) alloy cast nanocomposites fabricated by optimized mechanical milling and powder metallurgy techniques. Nanotechnol. Rev..

[37] Chohan J.S., Mittal N., Kumar R., Singh S., Sharma S., Dwivedi S.P., Saxena A., Chattopadhyaya S., Ilyas R.A., Le C.H. (2021). Optimization of FFF process parameters by naked mole-rat algorithms with enhanced exploration and exploitation capabilities. Polymers.

[38] Ilyas R.A., Sapuan S.M., Asyraf M.R.M., Dayana D.A.Z.N., Amelia J.J.N., Rani M.S.A., Norrrahim M.N.F., Nurazzi N.M., Aisyah H.A., Sharma S. (2021). Polymer composites filled with metal derivatives: A review of flame retardants. Polymers.

[39] Chohan J.S., Mittal N., Kumar R., Singh S., Sharma S., Singh J., Rao K.V., Mia M., Pimenov D.Y., Dwivedi S.P. (2020). Mechanical Strength Enhancement of 3D Printed Acrylonitrile Butadiene Styrene Polymer Components Using Neural Network Optimization Algorithm. Polymers.

[40] Singh Y., Singh J., Sharma S., Aggarwal V., Pruncu C.I. (2021). Multi-objective optimization of kerf-taper and surface-roughness quality characteristics for cutting-operation on coir and carbon fibre reinforced epoxy hybrid polymeric composites during CO2-pulsed laser-cutting using RSM. Lasers Manuf. Mater. Proc..

[41] Sharma S., Singh J., Kumar H., Sharma A., Aggarwal V., Gill A.S., Jayarambabu N., Kailasa S., Rao K.V. (2020). Utilization of rapid prototyping technology for the fabrication of an orthopedic shoe inserts for foot pain reprieve using thermo-softening viscoelastic polymers: A novel experimental approach. Meas. Control..

[42] Singh Y., Singh J., Sharma S., Sharma A., Chohan J.S. (2022). Process parameter optimization in laser cutting of coir fiber reinforced epoxy composite—A review. Mater. Today Proc..

[43] Chohan J.S., Kumar R., Singh T.H.B., Singh S., Sharma S., Singh J., Mia M., Pimenov D.Y., Chattopadhyaya S., Dwivedi S.P. (2020). Taguchi S/N and TOPSIS Based Optimization of Fused Deposition Modelling and Vapor Finishing Process for Manufacturing of ABS Plastic Parts. Materials.

[44] Prabhakaran S., Krishnaraj V., Sharma S., Senthilkumar M., Jegathishkumar R., Zitoune R. (2019). Experimental study on thermal and morphological analyses of green composite sandwich made of flax and agglomerated cork. J. Therm. Anal..

[45] Sharma S., Sudhakara P., Singh J., Ilyas R.A., Asyraf M.R.M., Razman M.R. (2021). Critical Review of Biodegradable and Bioactive Polymer Composites for Bone Tissue Engineering and Drug Delivery Applications. Polymers.

[46] Sharma S., Sudhakara P., Omran A.A.B., Singh J., Ilyas R.A. (2021). Recent Trends and Developments in Conducting Polymer Nanocomposites for Multifunctional Applications. Polymers.

[47] Jha K., Tyagi Y.K., Kumar R., Sharma S., Huzaifah M.R.M., Li C., Ilyas R.A., Dwivedi S.P., Saxena A., Pramanik A. (2021). Assessment of Dimensional Stability, Biodegradability, and Fracture Energy of Bio-Composites Reinforced with Novel Pine Cone. Polymers.

[48] Kadier A., Ilyas R.A., Huzaifah M.R.M., Harihastuti N., Sapuan S.M., Harussani M.M., Azlin M.N.M., Yuliasni R., Ibrahim R., Atikah M.S.N. (2021). Use of industrial wastes as sustainable nutrient sources for bacterial cellulose (BC) production: Mechanism, advances, and future perspectives. Polymers.

[49] Singh Y., Singh J., Sharma S., Lam T.-D., Nguyen D.-N. (2020). Fabrication and characterization of coir/carbon-fiber reinforced epoxy based hybrid composite for helmet shells and sports-good applications: Influence of fiber surface modifications on the mechanical, thermal and morphological properties. J. Mater. Res. Technol..

[50] Suriani M.J., Ilyas R.A., Zuhri M.Y.M., Khalina A., Sultan M.T.H., Sapuan S.M., Ruzaidi C.M., Wan F.N., Zulkifli F., Harussani M.M. (2021). Critical review of natural fiber reinforced hybrid composites: Processing, properties, applications and cost. Polymers.

[51] Kumar R., Ranjan N., Kumar V., Kumar R., Chohan J.S., Yadav A., Piyush, Sharma S., Prakash C., Singh S. (2021). Characterization of Friction Stir-Welded Polylactic Acid/Aluminum Composite Primed through Fused Filament Fabrication. J. Mater. Eng. Perform..

[52] Mohamed R.M., Yusoh K. (2015). A Review on the Recent Research of Polycaprolactone (PCL). Adv. Mater. Res..

[53] Azlin M.N.M., Ilyas R.A., Zuhri M.Y.M., Sapuan S.M., Harussani M.M., Sharma S., Nordin A.H., Nurazzi N.M., Afiqah A.N. (2022). 3D Printing and Shaping Polymers, Composites, and Nanocomposites: A Review. Polymers.

[54] Reis R.S., Souza D., de Holanda Saboya Souza D., de Fátima Vieira Marques M., da Luz F.S., Monteiro S.N. (2021). Novel bionanocomposite of polycaprolactone reinforced with steam-exploded microfibrillated cellulose modified with ZnO. J. Mater. Res. Technol..

[55] Kumar J., Singh D., Kalsi N.S., Sharma S., Pruncu C.I., Pimenov D.Y., Rao K.V., Kapłonek W. (2020). Comparative study on the mechanical, tribological, morphological and structural properties of vortex casting processed, Al-SiC-Cr hybrid metal matrix composites for high strength wear-resistant applications: Fabrication and characterizations. J. Mater. Res. Technol..

[56] Dwivedi S.P., Saxena A., Sharma S. (2021). Influence of Nano-CuO on Synthesis and Mechanical Behavior of Spent Alumina Catalyst and Grinding Sludge Reinforced Aluminum Based Composite. Int. J. Met..

[57] Muni R.N., Singh J., Kumar V., Sharma S. (2019). Parametric Optimization of Rice Husk Ash, Copper, Magnesium reinforced Aluminium Matrix hybrid Composite processed by EDM. ARPN J. Eng. Appl. Sci..

[58] Muni R.N., Singh J., Kumar V., Sharma S. (2019). Influence of rice husk ash, Cu, Mg on the mechanical behaviour of Aluminium Matrix hybrid composites. Int. J. Appl. Eng. Res..

[59] Dwivedi S.P., Saxena A., Sharma S., Srivastava A.K., Maurya N.K. (2021). Influence of SAC and Eggshell addition in the Physical, Mechanical and Thermal Behaviour of Cr reinforced Aluminium Based Composite. Int. J. Cast Met. Res..

[60] Saxena A., Dwivedi S.P., Dixit A., Sharma S., Srivastava A.K., Maurya N.K. (2021). Computational and experimental investigation on mechanical behavior of zirconia toughened alumina and nickel powder reinforced EN31 based composite material. Mater. Werkst..

[61] Sharma S., Singh J., Gupta M.K., Mia M., Dwivedi S.P., Saxena A., Chattopadhyaya S., Singh R., Pimenov D.Y., ErdiKorkmaz M. (2021). Investigation on mechanical, tribological and microstructural properties of Al-Mg-Si-T6/SiC/muscovite-hybrid metal-matrix composites for high strength applications. J. Mater. Res. Technol..

[62] Dwivedi S.P., Agrawal R., Sharma S. (2021). Effect of Friction Stir Process Parameters on Mechanical Properties of Chrome Containing Leather Waste Reinforced Aluminium Based Composite. Int. J. Precis. Eng. Manuf. Technol..

[63] Kumar J., Singh D., Kalsi N.S., Sharma S., Mia M., Singh J., Rahman M.A., Khan A.M., Rao K.V. (2021). Investigation on the mechanical, tribological, morphological and machinability behavior of stir-casted Al/SiC/Mo reinforced MMCs. J. Mater. Res. Technol..

[64] Aggarwal V., Singh J., Sharma S., Sharma A., Singh G., Parshad J. (2020). Empirical Modeling of Machining Parameters during WEDM of Inconel 690 using Response Surface Methodology. AIP Conf. Proc..

[65] Aggarwal V., Singh J., Sharma S., Garg H.K., Sharma A., Singh G., Parshad J. (2020). An experimental study of wire breakage frequency on different electrodes during WEDM of Inconel-722. IOP Conf. Ser. Mater. Sci. Eng..

[66] Aggarwal V., Pruncu C.I., Singh J., Sharma S., Pimenov D.Y. (2020). Empirical Investigations during WEDM of Ni-27Cu-3.15Al-2Fe-1.5Mn Based Superalloy for High Temperature Corrosion Resistance Applications. Materials.

[67] Qureshi M.N., Sharma S., Singh J., Khadar S.D.A., Baig R.U. (2020). Evaluation of Surface Roughness in the turning of Mild Steel under different cutting conditions using back propagation Neural Network. Proc. Est. Acad. Sci..

[68] Islam S., Dwivedi S.P., Dwivedi V.K., Sharma S., Kozak D. (2021). Development of Marble Dust/Waste PET Based Polymer Composite Material for Environmental Sustainability: Fabrication and Characterizations. J. Mater. Perform. Charact..

[69] Mamunya Y.P., Davydenko V.V., Pissis P., Lebedev E.V. (2002). Electrical and thermal conductivity of polymers filled with metal powders. Eur. Polym. J..

[70] Sharma S., Sudhakara P. (2019). Fabrication and optimization of hybrid AA-6082-T6 alloy/8%Al2O3(Alumina)/2%Grp metal matrix composites using novel Box-Behnken methodology processed by wire-sinking electric discharge machining. Mater. Res. Express.

[71] Singh H., Singh J., Sharma S., Dwivedi S.P., Obaid A.J. (2021). Comparative Performance of Copper, Graphite, Brass and Aluminium/Graphite Based Different Tool Electrodes for Optimizing the Material Removal Rate during Die-Sinking EDM of Stir-Casted, Al6061/SiC MMCs for Sustainable Manufacturing and Energy Applications. J. Green Eng..

[72] Dwivedi S.P., Saxena A., Sharma S., Singh G., Singh J., Mia M., Chattopadhyaya S., Pramanik A., Pimenov D.Y., Wojciechowski S. (2021). Effect of ball-milling process parameters on mechanical properties of Al/Al2O3/collagen powder composite using statistical approach. J. Mater. Res. Technol..

[73] Khare J.M., Dahiya S., Gangil B., Ranakoti L., Sharma S., Huzaifah M.R.M., Ilyas R.A., Dwivedi S.P., Chattopadhyaya S., Kilinc H.C. (2021). Comparative Analysis of Erosive Wear Behaviour of Epoxy, Polyester and Vinyl Esters Based Thermosetting Polymer Composites for Human Prosthetic Applications Using Taguchi Design. Polymers.

[74] Dwivedi S.P., Maurya M., Sharma S. (2021). Study of CCLW, Alumina and the Mixture of Alumina- and CCLW-Reinforced Aluminum-Based Composite Material with and Without Mechanical Alloying. J. Inst. Eng. Ser. D.

[75] Dwivedi S.P., Sahu R., Saxena A., Dwivedi V.K., Srinivas K., Sharma S. (2021). Recovery of Cr from chrome-containing leather waste and its utilization as reinforcement along with waste spent alumina catalyst and grinding sludge in AA 5052-based metal matrix composites. Proc. Inst. Mech. Eng. Part E J. Process. Mech. Eng..

[76] Dwivedi S.P., Maurya M., Saxena A., Sharma S. (2021). Synthesis and characterization of spent alumina catalyst and grinding sludge reinforced aluminium-based composite material. Proc. Inst. Mech. Eng. Part C J. Mech. Eng. Sci..

[77] Dwivedi S.P., Maurya M., Sharma S. (2022). Synthesis and characterisation of chromium, eggshell and grinding sludge-reinforced aluminium metal matrix composite: An experimental approach. Green Mater..

